# Cannibalism as a Possible Entry Route for Opportunistic Pathogenic Bacteria to Insect Hosts, Exemplified by *Pseudomonas aeruginosa*, a Pathogen of the Giant Mealworm *Zophobas morio*

**DOI:** 10.3390/insects9030088

**Published:** 2018-07-24

**Authors:** Gabriela Maciel-Vergara, Annette Bruun Jensen, Jørgen Eilenberg

**Affiliations:** 1 Department of Plant and Environmental Sciences, Faculty of Science, University of Copenhagen, Thorvaldsensvej 40, 3rd floor, 1871 Frederiksberg C, Denmark; abj@plen.ku.dk (A.B.J.); jei@plen.ku.dk (J.E.); 2 Laboratory of Entomology, Wageningen University, Radix Building 107, Droevendaalsesteeg 1, 6708 PB Wageningen, The Netherlands; 3 Laboratory of Virology, Wageningen University, Radix Building 107, Droevendaalsesteeg 1, 6708 PB Wageningen, The Netherlands

**Keywords:** entry route, disease transmission, opportunistic microorganism, bacterial infection, cannibalism, insect rearing

## Abstract

Opportunistic bacteria are often ubiquitous and do not trigger disease in insects unless the conditions are specifically favorable for bacterial development in a suitable host. In this paper, we isolated and identified a bacterium, *Pseudomonas aeruginosa*, from the larvae of the giant mealworm *Zophobas morio* and we studied the possible entry routes by challenging larvae with per os injection and subdermal injection. We also evaluated the effect of exposing groups of larvae to *P. aeruginosa* inoculated in their feed and the effect of exposing wounded larvae to *P. aeruginosa*. We concluded that the mortality rate of *Z. morio* larvae is higher when *P. aeruginosa* gets in direct contact with the hemolymph via intracoelomic injection compared to a situation where the bacterium is force-fed. Larvae with an open wound exposed to *P. aeruginosa* presented higher mortality rate compared to larvae with a wound that was not exposed to the bacterium. We documented too, that cannibalism and scavenging were more prevalent among larvae in a group, when *P. aeruginosa* is present compared to when it is absent. We discuss hereby different aspects related with the pathogen’s entry routes to insects the complexity of pathogen´s transmission in high population densities and different ways to prevent and/or control *P. aeruginosa* in mass rearing systems.

## 1. Introduction

Insect mass-rearing aims to obtain large numbers of individuals [1] in a limited space under controlled production conditions. Unfavorable production conditions such as high population density, high relative humidity, suboptimal temperature, non-balanced diet, and inbreeding are among the factors that may cause insects to suffer from physiological stress. Such stress increases the chance for disease outbreaks by opportunistic pathogens, and poses a challenge for insect rearing in terms of yield, productivity, and microbiological contamination (particularly in cases when insects are reared for human food or animal feed) [2,3]. Two well-known bacterial pathogens infecting insects, plants, and mammals are *Serratia marcescens* and *Pseudomonas aeruginosa* [4,5,6]. Both bacteria are ubiquitous [7], and they inhabit soil, fresh water, and the rhizosphere. Furthermore, they are able to live on other different surfaces. Their success when thriving in several environments is due to their ability to use different substrates and their conversion of many different sources into utilizable energy [8]. Particularly for *P. aeruginosa*, its prevalence in natural environments is enhanced by its ability to form biofilms that increase the growth surface and protects the bacteria from environmental factors (i.e., UV light, desiccation, etc.) [9]. They are opportunistic insect pathogens since they are usually present in low numbers inside or outside the insects without causing disease, but they may become pathogenic under certain stress conditions for the insects and/or when the insects have a weakened immune system [10]. *Serratia marcescens* is present as part of the gut microbiota of some sandflies and triatomine species [11]. Examples of *S. marcescens*, isolated as a pathogen from insects of the orders Coleoptera, Diptera, Orthoptera, and Lepidoptera, have been reported [12,13,14,15,16,17,18]. Similarly, *P. aeruginosa* has been isolated from different weevil species (the pecan weevil *Curculio caryae* [14]; the southern pine weevil *Dendroctonus brevicomis* [12]; the red palm weevil *Rynchophorus ferrugineus* [19], the tobacco and tomato hornworms *Manduca sexta* and *M. quinquemaculata* [20], from *Schistocerca gregaria* eggs [21] in the wild and from several other insect species. *P. aeruginosa* epizootic outbreaks have been reported in several insect species in laboratory rearings, but no epizootic event has been recorded in nature. High relative humidity combined with high temperature, are conducive factors for the development of *P. aeruginosa* in insects [7]. Virulence factors recognized for *P. aeruginosa* pathogenesis are the production of metalloproteases alkaline proteinase (aeruginolysin), elastase B (pseudolysin), elastase A (staphylolysin), and one serine proteinase. Also, the production of phospholipase C, proteinase IV, pili, flagella, exotoxins A, lipopolysaccharids (LPS), enzymes secreted by the type III secretion system, as well as quorum sensing (with its implications in biofilm formation) are also virulence factors [8,22]. For example, Exotoxin A has been associated with the inhibition of the synthesis of intracellular proteins [23]. A recent case of *P. aeruginosa* detection in large scale rearing occurred in a giant mealworm (*Z. morio*) production system in China [24]. *Zophobas morio* is one of three mealworm species that are being explored and mass reared as an alternative source of protein [25,26], and control of diseases is essential. At the end of 2015, we received in our laboratory a sample of *Z. morio* larvae from a commercial rearing colony with a high number of insects dying within a few days. We looked for pathogens in the larvae and we detected and isolated the bacterium *P. aeruginosa* in vitro. Our aim for the current study was to observe and describe the disease symptoms and to evaluate experimentally the different potential entry routes of the pathogen.

## 2. Materials and Methods

### 2.1. P. aeruginosa (Isolation, Identification and Preservation)

Infected larvae were obtained from a commercial insect production company. First, hemolymph from symptomatic alive larvae (immobilized prior to dissection for 10 min at −20 °C) and dead larvae (different times post mortem) was observed using light microscopy. Diseased and dead larvae were then surface sterilized (ethanol 70%; water; chlorine 3%, 1 min; water; ethanol 70%; water 2×) and dissected under sterile conditions. A 10 µL loop with hemolymph from individual larvae was streaked on nutrient agar (Standard I nutrient agar, Merck, Darmstad, Germany). Streaked plates were incubated at 32 °C for 48 h and thereafter verification of cell and colony morphology was done. A highly predominant type of colony was detected across the different streaked plates. Such type of colony was observed to be the only one growing particularly in plates streaked with hemolymph of symptomatic larvae and recently dead larvae (i.e., 24–48 h post mortem). Of these colonies, a single one was picked and thereafter inoculated in an Erlenmeyer flask (V = 250 mL) containing 30 mL of nutrient broth (Standard I nutrient broth, Merck, Darmstad, Germany) in an orbital shaker at 180 rpm and 32 °C for 24 h Cryopreservation vials were made by adding 20% *v*/*v* glycerol to the bacterial liquid culture. After confirmation of a gram negative bacterium, biochemical characterization was done using an API20E test strip (Biomeurieux, Grenoble, France) for gram negative bacteria. For molecular identification, 500 µL of bacterial liquid culture (cultured as described above) were pelleted at 5000 rpm for 5 min and 5 °C. The pellet was subjected to total DNA extraction. DNA extraction was done using the DNeasy^®^ Plant Mini Kit (Qiagen GmbH, Hilden, Germany). For PCR, 1 µL of DNA solution was added to a mix of 5 µL of each of the primers from the 16sRNA gene in bacteria (27F: AGAGTTTGATCMTGGCTCAG and 806R: GGACTACNNGGGTATCTAAT), 25 µL of premixed dNTp’s (TAKARA BIO, Shiga, Japan)) and Taq polymerase (AmpliTaq Gold polymerase & MgCl_2_, APPLIED BIOSYSTEMS, TermoFischer Scientific, Waltham, MA, USA) and 15 µL of water. The PCR program included in total 39 cycles of 15 s at 92 °C, 1 min at 55 °C and 50 s at 74 °C. PCR products were visualized on a 1% agarose gel and were purified with a GFX™ PCR DNA and Gel Band Purification Kit (GE Healthcare, Chicago, IL, USA). PCR products sequencing service was performed by Beckman Coulter Genomics (now Genewiz, South Plainfield, NJ, USA), using the same pair of primers used for DNA amplification. Alignment of the obtained sequence (Appendix A) and highly similar sequences (Megablast) with a maximum target of 100 sequences, was performed by using the software BLASTN 2.8.0+ (NCBI, Bethesda, MD, USA) [27].

### 2.2. Zophobas morio

Healthy larvae were purchased from a local retailer. Seventy larvae (~0.8–1 g) were placed individually in acrylic boxes for pupation. They were fed one carrot slice every 4 days and ad libitum diet (DT2) formulated at our laboratory (55.5% wheat bran, 25% oats, 16% brewer yeast, 3.5% casein). After eclosion, 50 non-sexed adults were randomly selected, combined in one container for mating to occur and reared to get a new generation of larvae. From this new generation, larvae of ~0.68–0.73 g (N = 54) were selected for *per os* injection and subdermal injection; larvae weighing ~0.07 g (N = 14 batches of 5 larvae; average larval weight was calculated) were used for the experiment of group exposure in the feed. Starting one day after injection, *Z. morio* larvae were fed every third day with a cube (~0.8 g) of agar diet (400 mL distilled water, 15 g DT2 and 10 g bacteriological agar) during and after the injection (*per os* and subdermal) and during and after the exposure by wounding. For the group exposure bioassay, one cube of agar diet was added on day 10 after the start of the experiment. All the experiments were incubated in darkness at 28 °C and at an average relative humidity of ~70%.

### 2.3. Infection Experiments

*P. aeruginosa* was cultured by inoculating 750 µL from a cryopreservation vial (prepared as described above) in an Erlenmeyer flask (V = 250 mL) containing 30 mL of nutrient broth (Standard I nutrient broth). The culture was incubated at 200 rpm for 20 h at 37 °C. Bacterial cells were washed three times (5000 rpm, 7 min, 5 °C) with a 10 mM MgSO_2_ solution in demineralized water. A bacterial suspension was prepared by adjusting OD_600_ (BioPhotometer, Eppendorf, Hamburg, Germany) to 0.7, equivalent to a concentration of ≈5.4 × 10^8^ cells/mL. Our bio-assay procedures, including the doses used in each type of experiments, were based on pilot experiments. The doses were chosen in order to address the research aims in the best way.

We conducted two bioassays in which purposeful injection of a specific dose was conducted, either per os (force-feeding) or subdermal. For these two experiments, larvae were treated and incubated individually. We also conducted one experiment in which groups of insects were exposed to *P. aeruginosa* cells in the feed. This experiment mimicked the natural wounding and scavenging that occurs in reared mealworms due to cannibalism. A last experiment was conducted in which *Z. morio* larvae were purposefully wounded and individually exposed to bacterial cells. Table 1 summarizes the experiments described above.

*Per os* injection: Force-feeding was performed by manually injecting 10 µL aliquots of a bacterial suspension to each of 20 larvae by using a blunt 0.32 gauge needle attached to a 100 µL syringe (Hamilton, Reno, NV, USA). Batches of 12 larvae were immobilized for 5 min at −20 °C prior injection. Two doses and a control were included in the experiment: a dose (D1) of approximately 1.1 × 10^7^ UFC/larva, a dose (D2) consisting of approximately half of the cells in D1 (D2 ≈ 5.4 × 10^6^ UFC/larva) and 10 mM MgSO_2_ solution as the control. Injection bioassays were done in triplicate. Mortality was recorded daily during 15 days.

**Figure 1:**
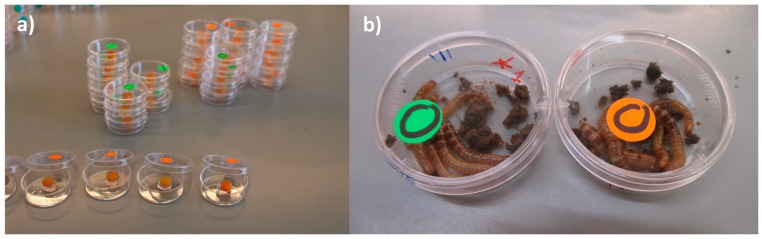
(**a**) Set-up of group exposure of larvae to *P. aeruginosa* (in the feed); (**b**) control larvae (green label) and larvae exposed to bacterial cells (orange label) 10 dpi.

Group exposure (in the feed): Five batches of 6 larvae (each larva weighed ~0.07 g) were placed each in a Petri dish (d = 3 cm, a = 5 cm^2^). Each batch was exposed as a group to a total load of ≈ 2.16 × 10^8^ (≈3.6 × 10^7^ UFC/larvae). A 24 µL droplet of a bacterial suspension of ≈9.0 × 10^9^ UFC/mL was deposited on a feeding arena made with a carrot disc (d = 0.8 cm; thickness ≈0.3 cm; weight ~0.16 g) that was placed on top of a 10 mM MgSO_2_ solution-agar disc in the middle of a Petri dish. The larvae of the control treatment were exposed to 24 µL of 10 mM MgSO_2_ (Figure 1a,b). The number of larvae chosen for this experiment was the maximum number of larvae that fitted in the petri dish area including the carrot-agar arena. We considered this setting to be representative of a high population density. The number of dead (categorized as visibly cannibalized or not) and missing larvae (due to cannibalism) was recorded daily during 15 days. The dead larvae were not removed as our aim was to mimic the densely populated mass-rearing conditions in which normally, removal of sick/dead individuals does not occur. In this experiment, it was not possible to determine the cause of dead for the larvae in each group (i.e., whether a larva died due to injuries caused by cannibalism and subsequent infection via the hemolymph, whether a weak (infected or not) larva was cannibalized before dead or whether a larva died due to *P. aeruginosa*’s infection and it was afterwards consumed by a conspecific larva). Hence, we expressed all three possible options as cannibalism and reported it as the difference between the total number of insects from the original group and that of the insects that remained at the end of the experiment. The group exposure experiment was done in triplicate.

Subdermal injection: Subdermal infection was performed by manually injecting 20 larvae with 10 µL aliquots of a bacterial suspension in the 2nd sternite (ventral) with a 45° beveled point 0.30 gauze needle attached to a 100 µL syringe (Hamilton, Reno, NV, USA). Two doses and a control were included in the experiment: a dose of approximately 5.4 × 10^6^ UFC/larva (D2: same as for *per os* injection), a dose which corresponds to approximately one tenth of D2 (D3 ≈ 5.4 × 10^5^ UFC/larva) and 10 mM MgSO_2_ solution as the control. Injection bioassays were done in triplicate. Mortality was recorded daily during 5 days.

Wounding: Wounding was done by abscission of the left hind leg tarsus of each of 20 larvae with sterile dissection scissors. One 20 µL droplet of a bacterial suspension with approximately 1.1 × 10^9^ UFC/mL (≈2.16 × 10^7^ UFC in total) was placed in the bottom of a Petri dish (d = 6 cm); a 20 µL droplet of a 10 mM MgSO_2_ solution was used as the control. A purposefully wounded larva was placed with the wound on top of the droplet. The experiment was conducted in duplicate. Mortality was checked daily for 10 days.

### 2.4. Statistical Analysis of Bioassays

Mortality values for the group of larvae injected per os (corrected by Henderson-Tilton’s formula) were distributed normally and subjected to One Way Analysis of Variance (ANOVA) at a significance level of 0.05, followed by a pairwise multiple comparison by the Student-Newman-Keuls test. Cannibalism rates in groups of larvae exposed (or not exposed) to *P. aeruginosa* were distributed normally and analyzed with a Student’s *t*-test at a significance level of 0.05. Data of mortality due to subdermal injection did not conform to the normal distribution so they were analyzed by a Krustal-Wallis test at a significance level of 0.05, followed by a pairwise multiple comparison by the Student-Newman-Keuls test. Mortality values of larvae exposed to *P. aeruginosa* by purposeful wounding were distributed normally and analyzed with a Welch’s *t*-test at a significance level of 0.05. All statistical analyses were performed using SigmaPlot v. 14 (Systat Software, San Jose, CA, USA).

## 3. Results

### 3.1. Observations on Disease Symptoms in Z. morio Larvae

**Figure 2:**
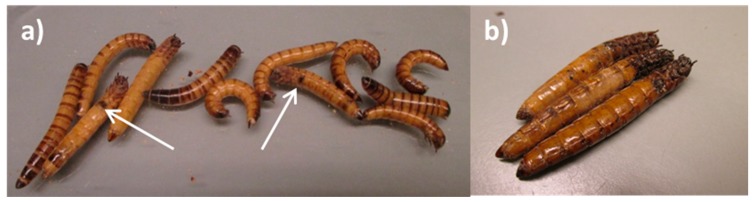
(**a**) Diseased *Z. morio* larvae displaying melanized stains before death (white arrows); (**b**) *Z. morio* larvae in a process of melanization, 48 h after death.

Diseased larvae received from the insect producer ceased feeding and curved their bodies with a ventral orientation, almost entirely losing their mobility. This position could last for 2–4 days before the insects died, however close to the death the insect body became flaccid most of the times. Molting frequency seemed lower for diseased larvae compared to healthy larvae. Cannibalism of diseased larvae was observed, often in the legs’ region. Melanization started with small stains which increased in size during the 72 h after the first stain appeared. Melanization eventually took over parts of or even most of the body in moribund larvae, as a result of a septicemia and lysis of the larval inner organs and tissues, resulting in the characteristic dark brown colored larvae. Dead larvae turned black, especially towards each end (Figure 2a,b). An unpleasant odor was characteristic for moribund and dead larvae. For larvae where melanization was absent, the gut remained undisrupted. In those where melanization started, the gut was in a phase of disintegration.

### 3.2. Characterization of P. aeruginosa

**Figure 3:**
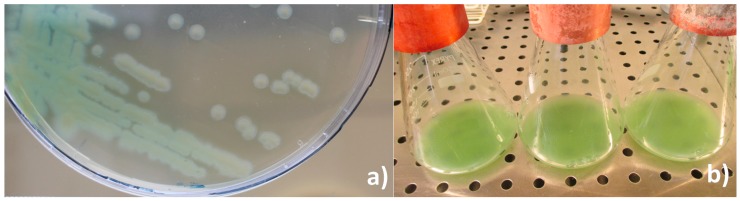
(**a**) In vitro solid (nutrient agar) and (**b**) liquid culture (nutrient broth) of *P. aeruginosa* isolate from *Z. morio* larvae.

Colony morphology and in vitro cultivation: By direct observation of the hemolymph from diseased larvae, the presence of bacilliform non-sporulated, non crystalliferous bacterial cells was confirmed. In vitro cultivation in nutrient agar resulted in just one type of colonies: greenish, convex but flattened as growth progressed, round with slight irregular borders and slimy (Figure 3a), suggesting a monospecific infection. In vitro liquid cultivation in nutrient broth resulted in a yellow-green pigmented culture that changed color to green-blueish when agitated (Figure 3b).

Biochemical characterization Comparison of the biochemical tests was done using corresponding values for reference strains of *P. aeruginosa* and *Pseudomonas fluorescens*/*putida* reported in the API database (Table 2).

Molecular identification: The length of the amplified 16s RNA gene fragment for the our bacterial isolate was 770 bp. Out of 100 hits, 95 sequences produced significant alignments with different accessions of *P. aeruginosa*, including sequences of the 16S ribosomal RNA gene and also sequences of the whole genome of *P. aeruginosa* (i.e., NCBI—reference strains PAO1 and PA14; Accession numbers: NC_002516AE00409). The query cover for the alignment was 100% as well as the sequences identity, with a total maximum score of 1423 and a 735 bp effective length of the query.

### 3.3. Infection Experiments

**Figure 4:**
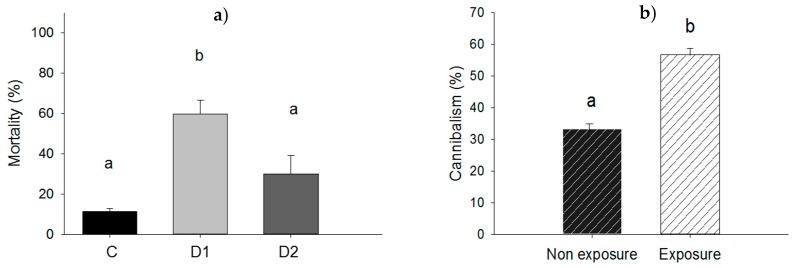
(**a**) Mortality rate of larvae force-fed with cells of *P. aeruginosa* at doses D1 of ≈1.1 × 10^7^ UFC/larva (D1), D2 of ≈5.4 × 10^6^ UFC/larva (D2) or 10 mM MgSO_4_ solution as a control (C); (**b**) cannibalism rate among larvae in groups exposed to ≈2.2 × 10^8^ UFC of *P. aeruginosa*. Bars having no letters in common differ significantly (*p* ≤ 0.05). Error bars indicate the standard error of the mean.

**Figure 5:**
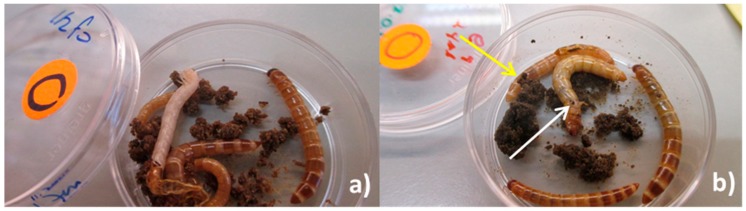
(**a**) Larvae molting in group exposure experiment; (**b**) injury due to cannibalism in larva’s proleg (white arrow) and cannibalized dead larva (yellow arrow).

Force-feeding of larvae with *P. aeruginosa* resulted in higher mortality at the dose D1 when compared to dose D2: 59.6% vs. 30.0%; *p* = 0.020 and significantly higher than mortality in control (*p* = 0.005). However, mortality was not significantly different for the control and dose D2 (11.3% vs. 30.0%; *p* = 0.096) as shown in Figure 4a. For the experiment in which larvae were exposed to *P. aeruginosa* in groups, there were no dead larvae at the end of the experiment and cannibalism rate was compared in exposed and non-exposed groups of larvae (Figure 5a,b). Amongst larvae exposed in groups to ≈2.2 × 10^8^ UFC, cannibalism rate was 56.6% compared to 33.3% for unexposed control larvae (*p* = 0.001) (Figure 4b).

**Figure 6:**
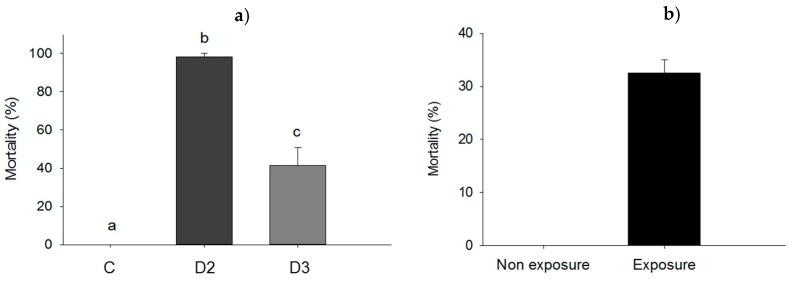
(**a**) Mortality rate of larvae injected subdermal with either dose 2 (D2) of ≈5.4 × 10^6^ UFC/larva), dose 3 (D3) of ≈5.4 × 10^5^ UFC/larva) of *P. aeruginosa* cells, or 10 mM MgSO_2_ solution as a control (C); (**b**) mortality of *Z. morio* larvae subjected to *P. aeruginosa* exposure by wounding. Bars having no letters in common and differ significantly (*p* ≤ 0.05). Error bars indicate the standard error of the mean.

Subdermal injection without bacteria did not affect larval survival (0% mortality rate for control). However, the mortality rate of larvae subjected to subdermal injection with dose 2 (D2) was significantly higher (98.3%; *p* = 0.05) compared to the mortality rate of larvae injected with dose 3 (D3) (41.3%; *p* = 0.05) and the control (Figure 6a). Wounding in itself was found not to influence mortality (0% in the control) whereas wounding and exposure to ≈2.2 × 10^8^ cells of *P. aeruginosa* resulted in a mortality rate of 32.5% (*p* = 0.049) (Figure 6b).

## 4. Discussion and Conclusions

The bacterium isolated from larvae of the giant mealworm *Z. morio* was identified as *P. aeruginosa*, based on the following well-known physical and biochemical characteristics (colony morphology, culture medium affinity, culture color) and ultimately by molecular tools. A relevant characteristic was siderophore chromatogenesis, a key feature for members of the *Pseudomonas* genus [28], also confirmed by the green-yellowish color of the in vitro solid and liquid cultures. Siderophores pyocianin and pyoverdin are essential virulence factors of *P. aeruginosa* during the infection process in animal species [29,30]. 

Diseased *Z. morio* larvae exhibited general symptoms that are described for infection of other insect species with *P. aeruginosa*: weakness, sluggishness, cease of feeding, melanization, putrid odor, and finally, death [19,21]. Importantly, the first symptoms of infection caused by *P. aeruginosa* can go unnoticed, and in several cases, only when insects are moribund and start to die, the disease becomes apparent [21,23]. *P. aeruginosa* is normally present in small numbers and can cause disease if it enters the insect hemocoel by conducive factors, which weaken the insects by means of different physiological stressors. Most bacteria enter the insect hemocoel by an injury or aided by a “support host factor” (i.e., gut pH) [23]. Regardless of the entry route, the wide range of *P. aeruginosa* virulence factors allows the bacterium to induce disease in insects, if conditions for such infections are favorable.

The mechanism of *P. aeruginosa* causing disease after getting in contact with the hemolymph is well known, while the mechanisms by which ingestion of a high number of *P. aeruginosa* cells can overcome the gut barriers and reach the insect hemocoel are still poorly understood. It is documented that as few as 10 to 20 cells of *P. aeruginosa* are enough to kill a grasshopper when injected directly into the hemocoel, compared to a wide range of 800 to 29,000 cells when incorporated in the feed (Table 3). In our study, mortality rates due to subdermal infection occurred as early as reported in the literature for other insects, though the doses we used were higher than those used in similar studies (Table 3). Our isolate of *P. aeruginosa* caused lower mortality of *Z. morio* larvae at the same time post-infection and overall when injected *per os*, compared to when injected directly into hemocoel at the same dose. These results are in accordance with studies on mortality rates in other insect species subjected to *P. aeruginosa*. In such studies, LD_50_ values and effective doses are much smaller for hemolymph injection compared to force-feeding or incorporation in the feed [23]. It is reported that regardless of the initial dose, *P. aeruginosa* reaches concentrations of 1 × 10^8^ to 1 × 10^9^ cells just before the host’s death for some insect species such as grasshoppers, the great wax moth *Galleria mellonella*, and the yellow mealworm *Tenebrio molitor* [21,23]. Looking at our own results, the overall mortality rate of larvae force-fed with *P. aeruginosa* was relatively high a very short time post infection (24 h). Hence, we hypothesize that a dose of ≈1.1 × 10^7^ UFC/larva would be high enough to allow bacterial reproduction and toxin synthesis within one day and would allow bacteria to be able to trespass the midgut epithelium and spread into the hemocoel. In this regard, it is reported that the pilli and flagella of *P. aeruginosa* act as adhesins by binding to a host cell receptor, establishing an essential linkage for the persistence and spread of *P. aeruginosa* by epithelial cell invasion/infection and cytotoxicity [8]. Thus, the dose of the initial inoculum of *P. aeruginosa* that enters an insect body is an important determinant of disease development in terms of intensity (post infection bacterial load) and speed (days post infection until death) [23].

Our results from the group exposure to *P. aeruginosa* exemplify how an opportunistic bacterial pathogen may affect production systems where insects are reared at high population densities during part or the whole of their life cycles, such as *Z. morio*. We found that the cannibalism rate was 23% higher for the groups exposed to *P. aeruginosa*, compared to the non-exposed groups of larvae, supporting the evidence of cannibalism as a significant enabler for insect pathogens to be transmitted [36,37]. Cannibalism is a common behavior, documented for quite a number of insect species [38]. Cannibalism often affects individuals that are smaller, weaker or more vulnerable, especially, at high population densities where food and space competition are more likely to occur. Molting and pupation are the susceptible stages for cannibalism to occur in mealworms, particularly for *Z. morio* larvae, for which a higher risk for pathogen transmission rate exists due to the fact that pupation is inhibited by crowding. Molting can be prolonged indefinitely until larvae die of old age if they do not find the chance to disperse as individuals, in order to pupate [39,40]. At the same time, our own experiments showed that ingesting a relatively high amount of bacterial cells (≈1.1 × 10^7^ UFC/larva) killed roughly 60% of the larvae, implying larvae may get weakened after the ingestion of bacteria, becoming more susceptible to be cannibalized upon, becoming a source for subsequent infection of other larvae. In this regard, transmission takes place because besides cannibalism, which occurs to living individuals, there is the added effect of scavenging or conspecific necrophagy, which implies feeding on dead (infected) larvae. 

On the same line, our experiment of purposeful wounding (average mortality rate = 32%) suggests that if larvae get accidentally or purposefully injured by a conspecific individual, a pathogen present in the environment may rapidly infect the insects. It has been reported that dead grasshoppers, due to *P. aeruginosa infection*, harbored a load of 2.4 × 10^10^ bacterial cells [21], which could potentially contaminate directly the substrate and serve as a concentrated inoculum for progressive bacterial growth on the substrate and frass. 

Not every insect species reared under high population density is as susceptible to pathogens [41,42] as mealworms, however, there are other factors that can contribute to infection by opportunistic bacteria such as *P. aeruginosa*: mixed infections as those occurring to other host-pathogen complexes [43,44,45,46], inbreeding [47,48], nutritional imbalance [49,50], and the relation between temperature and relative humidity [6,51,52,53]. 

## 5. Conclusions

We concluded that *P. aeruginosa* is lethal for *Z. morio* larvae at specific doses when injected intracoelomically and *per os*. Mortality rate is higher for wounded larvae that are exposed to *P. aeruginosa* compared to wounded larvae that are not exposed. We concluded too that the transmission of an opportunistic pathogen as *P. aeruginosa* at a high host population density is the result of the interaction of different factors. *Pseudomonas aeruginosa*’s transmission is enhanced by cannibalism, added the effects of scavenging. Insect behaviors that rarely occur in nature for some insect species may therefore be of high significance in commercial insect rearing, and it is a challenge for the insect industry to manage insect pathogens benefiting from cannibalism. *P. aeruginosa* is an example of one such bacterium whose prevention is needed since it may be detrimental for insect production. Contact with high dosages should be avoided for the staff (from a working environments’ perspective), since the bacterium is opportunistic. To prevent or manage *P. aeruginosa*, several actions must be taken. First, moribund and dead individuals must be scouted and removed immediately and treated as microbial waste before being discarded (i.e., by pyrolysis). Second, insect feed and the equipment used must be checked for presence of bacterial contamination and sterilized.

Additional actions that should be taken include the following: (a) to verify that the temperature of the water for washing trays and other equipment is around 50 to 60 °C; (b) to include a “soaking step”, where the rearing trays and other plastic materials remain for some minutes in a solution containing an EU approved disinfectant (highly advisable if contains ethylene diamine tetra acetic acid EDTA, due to its ability to reduce the formation of biofilms in surfaces of bacteria like *P. aeruginosa*); and (c) to reduce the relative humidity in the rearing and in the substrates (below ~60%) as *P. aeruginosa* thrives well in high relative humidity and it is highly sensitive to desiccation.

As a final remark, it is important to mention that in insect rearing, the production system must be evaluated and eventually redesigned to avoid stressed and weakened insects and to minimize the risk of cannibalism.

## Figures and Tables

**Table 1:** Summary of experiments conducted in this work.

	Possible Entry Route/Transmission Route	
Experiment	*Per os*	Cuticular
Force-feeding	x	
Group exposure (feed)	x	x
Subdermal injection		x
Purposeful wounding	x	x

**Table 2:** Biochemical characterization of the *P. aeruginosa* isolate from *Z. morio* by the API20E test.

Microorganism/Reaction	ONPG	ADH	LDC	CIT	H_2_S	URE	TDA	IND	VP	GEL	GLU	MAN	INO	SOR	RHA	SAC	MEL	AMY	ARA	OX	NO_2_
Isolate from *Z. morio*		X		X			X			X	X								X	X	
*P. aeruginosa*		X		X		X				X	X						X		X		
*P. fluorescens/putida*		X		X					X	X	X						X		X	X	

ONPG = beta-galactosidase; ADH = L-arginine dihydrolase; LDC = lusine decarboxylase; CIT = trisodium citrate utilization; H_2_S = H_2_S production; URE = urea hydrolysis; TDA = deaminase; IND = indole production; VP = acetoin production; GEL = gelatinase; GLU = glucose fermentation/oxidation; MAN = mannitol fermentation/oxidation; INO = inositol fermentation/oxidation; SOR = sorbitol fermentation/oxidation; RHA = rhamnose fermentation/oxidation; SAC = sucrose fermentation/oxidation; MEL = melibiose fermentation/oxidation; AMY = amygdalin fermentation/oxidation; ARA = arabinose fermentation/oxidation; OX = oxidase.

**Table 3:** Mortality rate of different insect species challenged with *P. aeruginosa* by different methods.

Order	Insect Species	Instar	Force-Feeding			Diet Incorporation			Sub Dermal Injection			Other Method			Reference
Entry Route			Per os			Per os			Cuticular			Cuticular			
			Dose (UFC/ind.)	Mort (%)	DDpi *	Dose (UFC/ind.)	Mort (%)	DDpi *	Dose (UFC/ind.)	Mort (%)	DDpi *	Dose (UFC/ind.)	Mort (%)	DDpi *	
**Lepidoptera**	*Galleria mellonella* (1)	L5							1	50	2+				[6,31]
	*Bombyx mori* (2) (3)	L5							3.57 × 10^2^	60	2				[32]
		L5							3.57 × 10^4^	100	2				
**Orthoptera**	*Camulla pellucida & Melanoplus bivitattus* (4)	-				8 × 10^3^–2.9 × 10^3^	50	7–21	10–20	50	2–3				[21]
	*Schistocerca gregaria* (5)	-				1.8 × 10^4^–2.4 × 10^4^	50	2	6 × 10^4^	100	1	5.3 × 10^4^ (°)	100	1	[33]
												5.3 × 10^4^	100	2	
**Diptera**	*Drosophila melanogaster* (6)	Adult							-	50	1+				[34]
	*D. melanogaster* (2) (7)	Adult							-	100	1+				[35]
**Coleoptera**	*Rynchophorus ferrugineus* (8)	L1	1 × 10^5^	50					9 × 10^2^	50	6 ± 3	1 × 10^5^	50	8 ± 3	[19]
		L2	1 × 10^5^	50					1 × 10^3^	50	6 ± 3	1 × 10^5^	50	8 ± 3	
		PP	4 × 10^5^	50					2 × 10^3^	50	6 ± 3	2 × 10^5^	50	8 ± 3	
	*Z. morio* (9)	L	1.1 × 10^7^	56	1–2				5.4 × 10^6^	98	1–2	2.2 × 10^7^ (^)	32	11–12	This work
		L	5.4 × 10^6^	30	1–2				5.4 × 10^5^	41	4–5	3.6 × 10^7^ (^^)	56.6	15	

* Ddpi indicates death at days post-infection (1) *P. aeruginosa* PA14. Mortality values correspond to the calculated median lethal dose (LD50). (2) *P. aeruginosa* PA01. (3) Calculated LD50 = 2.26 ± 0.03 × 10^2^. (4) There is no indication of which was the specific species used for each infection method. (5) Mortality value due to diet incorporation corresponds to the calculated LD50. Other infection method: spraying. (°) The difference in time (to reach 100% mortality) between the two spraying experiments was probably due to the incubation conditions: DDpi 1 = 38 + 2 C and 35 + 5% RH and DDpi 2 = 25 + 2 C and 85 + 5% RH. (6) No exact dose was injected. Flies were pricked in the thorax with a needle the tip of which was dipped in suspension of *P. aeruginosa* (ATCC25102) calibrated at 0.45 OD600/mL, without indication of the UFC concentration. (7) 4–7-d old male flies. Infection by pricking flies on the dorsal thorax with a needle dipped into a cell suspension containing 107 CFU. (8) Mortality values correspond to calculated LD50. Larval weight: L1, small larvae = 1.9 + 0.7 g; L2, large larvae = 4.3 + 1.4 g; PP, pre-pupae = 5.7 + 1.4 g. Other infection method: wading. (9) Larval weight: 0.8 + 0.1 g. Other infection method: wounding (^) and group exposure (^^). For the group exposure experiment, mortality rate is the result of cannibalism.

## Appendix A. FASTA Sequence of *P. aeruginosa* Isolate from *Z. morio*

ATGGCTCAGATTGAACGCTGGCGGCAGGCCTAACACATGCAAGTCGAGCGGATGAAGGGAGCTTGCTCCTGGATTCAGCGGCGGACGGGTGAGTAATGCCTAGGAATCTGCCTGGTAGTGGGGGATAACGTCCGGAAACGGGCGCTAATACCGCATACGTCCTGAGGGAGAAAGTGGGGGATCTTCGGACCTCACGCTATCAGATGAGCCTAGGTCGGATTAGCTAGTTGGTGGGGTAAAGGCCTACCAAGGCGACGATCCGTAACTGGTCTGAGAGGATGATCAGTCACACTGGAACTGAGACACGGTCCAGACTCCTACGGGAGGCAGCAGTGGGGAATATTGGACAATGGGCGAAAGCCTGATCCAGCCATGCCGCGTGTGTGAAGAAGGTCTTCGGATTGTAAAGCACTTTAAGTTGGGAGGAAGGGCAGTAAGTTAATACCTTGCTGTTTTGACGTTACCAACAGAATAAGCACCGGCTAACTTCGTGCCAGCAGCCGCGGTAATACGAAGGGTGCAAGCGTTAATCGGAATTACTGGGCGTAAAGCGCGCGTAGGTGGTTCAGCAAGTTGGATGTGAAATCCCCGGGCTCAACCTGGGAACTGCATCCAAAACTACTGAGCTAGAGTACGGTAGAGGGTGGTGGAATTTCCTGTGTAGCGGTGAAATGCGTAGATATAGGAAGGAACACCAGTGGCGAAGGCGACCACCTGGACTGATACTGACACTGAGGTGCGAAAGCGTGGGGAGCAAACAGGATTAGATA
